# Parents’ Growth Mindsets and Home-Learning Activities: A Cross-Cultural Comparison of Danish and US Parents

**DOI:** 10.3389/fpsyg.2020.01365

**Published:** 2020-07-08

**Authors:** Laura M. Justice, Kelly M. Purtell, Dorthe Bleses, Sugene Cho

**Affiliations:** ^1^Crane Center for Early Childhood Research and Policy, The Ohio State University, Columbus, OH, United States; ^2^Trygfonden Centre for Child Research, Aarhus University, Aarhus, Denmark

**Keywords:** growth mindset, home-learning environment, mindset, cross-culture, preschool

## Abstract

Mindset is a term commonly used to represent an individual’s beliefs about the role of ability and effort in learning. In this study, we assessed parental mindset—ability mindset and effort mindset—for 497 parents in two countries (United States and Denmark), all of whom had at least one child between 3 and 5 years of age. Of primary interest was assessing the relations between parental mindset and home-learning activities of four types: family learning activities, learning extensions, parental time investment, and parental school involvement. Findings showed that parents in the United States and Denmark held similar ability and effort mindsets, but differed significantly in home-learning activities, with US parents providing significantly more family learning activities, learning extensions, and parental time investment than Danish parents, although the latter had significantly higher levels of school investment. Furthermore, findings showed that parents’ effort mindset was a significant predictor of family learning activities and parental time investment and that country moderated the relations between effort mindset and parental time investment. For US parents, higher levels of effort mindset were associated with higher levels of parental time investment, but this was not the case for Danish parents. We call for experimental work to determine the causal relations between parental mindset and home-learning activities, and rigorous cross-cultural research to explore the universality of parental mindset in distinctive cultural settings.

## Introduction

The experiences we have in early childhood profoundly shape the rest of our lives (Duncan et al., 2010; Evans and Kim, 2013). In light of this, researchers have sought to develop and test interventions targeting this critical developmental period, to include interventions that enhance parents’ behaviors toward their children (Fishel and Ramirez, 2005; Roberts and Kaiser, 2011). However, changing the behaviors of parents can be notoriously challenging (Wagner et al., 2002). One potential reason for this is that parents’ beliefs about their children, and their own roles in shaping their children’s development, may be incompatible with the goals of these interventions. For instance, parents may be reluctant to talk frequently with their very young children, a frequently targeted goal in parent-focused early intervention activities (e.g., Lederer, 2001) if they do not understand that this is a mechanism through which language development occurs (Rowe, 2008).

Recent research has focused on parents’ mindsets as a key factor that may shape parenting behaviors and potentially moderate the success of parent-focused interventions (e.g., Rowe, 2008). Parents’ mindsets refers to the beliefs that caregivers hold regarding whether their children’s development is fixed or malleable as well as the importance of effort for learning (Sisk et al., 2018). There is currently great interest in exploring whether modifications to parents’ mindsets might influence their children’s development. For example, a recent large-scale intervention in Denmark found that providing parents with information about the malleability of children’s reading skills, in combination with reading materials, led to sizeable effects on children’s reading and writing skills, and these effects were largest when parents held fixed beliefs about the malleability of language skills prior to intervention (Andersen and Nielsen, 2016). More recently, Rowe and Leech examined use of a parent mindset intervention to improve parents’ non-verbal interactions with their 10-month-old infants (Rowe and Leech, 2018). Parents assigned to a treatment condition participated in training emphasizing the malleability of early language skills and that parents can play a key role in facilitating these skills through non-verbal interactions. Compared to parents in a control group, the trained parents non-verbally interacted more frequently with their children at 12 months of age, and this effect was particularly strong for parents with fixed mindsets at baseline, similar to the result reported by Andersen and Nielsen (2016). Such work suggests that parental ability mindsets may be a key target for interventions aiming to improve children’s early experiences and development, and should be incorporated into parent-focused interventions.

Yet, empirical understanding of the mindsets of parents with young children is still only emerging, and there is a need to increase knowledge about the variability among caregivers in their mindset toward their children, in particular the role that mindset may play in fostering children’s development. The present study was designed to increase our knowledge of parental mindset toward their preschool-aged children, to include assessing the extent to which parents’ ability and effort mindsets were associated with home-learning activities with their children, and to do so in two cultures—the United States and Denmark. As we will discuss, these two cultural contexts differ significantly in terms of societal inequality, availability of social benefits, and the frequency of “dual earner” couples; we speculated that parental mindset and time investments with their children may vary across these distinct cultural contexts. To our knowledge, this is the first cross-cultural investigation of how parents view their role in their children’s lives using a mindset lens, and results may help to inform future interventions designed to improve the developmental experiences of young children.

### Learning in the Home Environment and Parental Mindset

Considerable evidence shows that the home-learning environment provides young children with key opportunities to develop skills in a variety of domains, including early literacy, language, and numeracy (Breit-Smith et al., 2010; Manolitsis et al., 2013; Niklas et al., 2016). However, there is also significant variability in the extent to which young children experience those opportunities that facilitate early learning in the home environment. For instance, a study of the home-learning environment for children in Israel showed distinct differences in the volume of key experiences for children from lower-socioeconomic status (SES) homes compared to children from higher-SES homes: the former had significantly fewer educational games and books in the home and were read to less often than the latter group of children (Korat et al., 2007). Some evidence suggests that SES may represent a proxy for caregivers’ attitudes and knowledge about the importance of home-literacy opportunities, as more highly educated parents report higher regard for the importance of reading to their children and their own roles in facilitating early skill development than less-educated parents. In turn, such attitudes are positively correlated with the volume of learning opportunities provided to children in the home (Curenton and Justice, 2008). There are other reasons, however, that may explain variability in the home-learning experiences of young children, such as children’s gender (Baker and Milligan, 2016) and parent employment outside of the home (Heiland et al., 2017). For the latter, some evidence suggests that time investments in children decline as a function of maternal employment: as maternal work hours increase, there is an inverse effect on the time parents directly invest in their children (Heiland et al., 2017).

In the present study, we build upon such work by examining the extent to which caregiver mindsets—in particular, their mindset regarding the malleability of their children’s skills and perceptions regarding the importance of effort—were associated with home-learning activities, to include time investments. We examined four dimensions of home-learning activities, namely, family learning activities, learning extensions, parental time investments, and parental school involvement. *Family learning activities*, the most common aspect of the home-learning environment studied (e.g., Curenton and Justice, 2008), represent concrete activities caregivers do in the home with their children, such as telling stories and sharing books. Studies typically find a moderate association between the frequency of family learning activities and children’s early skills (Korat et al., 2007), with structural models (e.g., Christian et al., 1998) and experimental studies (e.g., Justice et al., 2011) supporting causal relations between these activities and children’s skill development. *Learning extensions* represent activities that might enhance children’s skills beyond the immediate home environment, such as visits to libraries and bookstores. Evidence suggests that such learning extensions is a distinct factor of the home-learning environment (Gonzalez et al., 2011) and serves to differentiate clusters of families in the nature of home-learning opportunities (Davis et al., 2016). *Parenting time investments* represent the actual amount of time parents report directly engaging with their children. Parents vary significantly in the amount of time they report to spend with their children (Kalil et al., 2014), and these differences are associated with household SES, family structures, and child birth order (Neidell, 2000; Mammen, 2011), as well as children’s early and later skill development (Bono et al., 2016). Finally, *parental school involvement* reflects the extent to which parents are engaged in their children’s early schooling. Research consistently finds positive effects of parents’ involvement in their children’s schools, including actual engagement in school-based activities as well as ongoing communication with children’s teachers, and is positively associated with children’s short- and longer-term academic development (Dearing et al., 2008; Bono et al., 2016).

For these four dimensions of the home-learning environment, we examined interrelations with parent mindset as measured across two dimensions, which we refer to as ability mindset and effort mindset. Both dimensions serve to capture variability in an individual’s implicit theories of intelligence and ability (Blackwell et al., 2007). *Ability mindset* captures parents’ beliefs regarding the fixedness of children’s skills, which may range along a continuum reflecting, on one end, the perspective that children’s skills are fixed/stable to and, on the other end, the perspective that children’s skills are malleable (Sisk et al., 2018). A parent with a fixed mindset would tend to believe that her child cannot improve, for instance, her reading skills, even with considerable work, as reading skills are fixed, whereas a parent with a growth-oriented ability mindset would believe that her child can significant improve her reading skills with work. *Effort mindset* captures parents’ beliefs regarding the specific importance of effort as an impetus to their children’s learning; parents with high effort beliefs perceive that hard work leads to greater learning (and thus place value on hard work), relative to parents with low effort beliefs, who would perceive that working hard is irrelevant or unimportant as it cannot override stable skills. Often, investigations of one’s perspectives about the role of effort in skill development has focused on adolescents (e.g., Blackwell et al., 2007), although in this study, we considered parental perspectives as to whether their children’s effort can contribute to early learning as an augment to focusing specifically on mindset.

### Parental Mindset and Early Learning: Cross-Cultural Considerations

The present study was designed to examine variability in parental mindset across two dimensions (ability mindset and effort mindset) and also the interrelations among mindset and home-learning activities, including parental time investment in their children. We also sought to explore whether this phenomenon may vary cross-culturally, potentially due to cultural conditions that contribute to variability in parental time with their children as a function of universal childcare. Here, we consider two countries—the United States and Denmark—which vary substantially in the degree of societal inequality, as a function in part of welfare provision as a means to reduce inequality, including universal childcare. In countries with considerable equality in income distribution, the Gini index is low, whereas in countries with considerable inequality in income distribution, the Gini index is high. Based on data from the Central Intelligence Agency collected across the 2000s and 2010s, countries in the lowest quintile for inequality, based on the Gini index (<31), are almost uniform in Europe (e.g., Denmark, France, Germany, Iceland, and Norway), whereas those in the highest quintile (>46) are largely in Africa (e.g., Botswana, Namibia, Rwanda, and Zambia) and Latin America (e.g., Chile, Colombia, Guatemala, Nicaragua, and Panama) (Central Intelligence Agency, 2020). The United States is in the second highest quintile (Gini index = 45), with inequality similar to that reported for Guanaya and Thailand (44.6), Peru (45.3), and Mozambique (45.6). One avenue for reducing income disparities is the provision of social welfare, including universal childcare to caregivers with young children.

For the present study, we conducted cross-cultural comparisons of parental mindset and home-learning activities for parents in the United States and Denmark; these countries’ Gini indices were 41.4 (2016) and 28.7 (2017), respectively (World Bank, 2020). This allows a comparison of a setting with limited investment in childcare versus a near-universal system of care. Both countries are in the 36-member Organisation for Economic Co-operation and Development (OECD) and represent the lowest level of inequality among members (Denmark) and among the highest (United States) based on a recent report from the OECD (Organisation for Economic Co-operation and Development, 2015). An additional important distinction between these settings concerns parent involvement in the labor market and the provision of numerous supports in welfare states to enable labor-force participation, including universal childcare (Sayer and Gornick, 2012). In turn, this can affect caregivers’ time investments with their children; for instance, Danish partnered mothers spend about 3 h less time with their children compared to their counterparts in the United States (Sayer and Gornick, 2012).

We might speculate that variabilities in the provision of universal child-care and labor-force participation patterns that distinguish these two cultures may contribute to differences in parental mindset and home-learning activities. Drawing from Bronfenbrenner’s ecological theory concerning the impacts of environment on child development (Bronfenbrenner, 1994), societal inequality and welfare access represent an important characteristics of the exosystem in which parents are rearing their children and children are developing and which may influence parenting behaviors and children’s development. In terms of the more proximal microsystem, access to a strong system of social supports, as is available in welfare-based societies, caregivers may have more positive perceptions of their own role in shaping their children’s development and may provide enhanced early-learning experiences for their children (Armstrong et al., 2005). Nonetheless, it is also the case that, in socialized economies like Denmark, where early childhood care and education is heavily subsidized with capped monthly fees and income-related sliding scales, there is far higher labor-force participation by mothers (more than 80% of mothers with children aged 0–14 are in work, which is the second highest in OECD; Organisation for Economic Co-operation and Development, 2020a), and higher volumes of time spent working by adults (more than 70% of couples are “full-time dual earner” couples which is the highest in OECD), which may detract from parents’ opportunities to provide home-learning activities for their children, irrespective of mindset.

### Study Aims

The overarching goal of this study was to examine how parental mindset may be associated with caregivers’ engagement in home-learning activities, specifically across distinct cultural contexts: the United States and Denmark. First, we conducted cross-cultural comparisons of the level of parental mindset and home-learning activities by comparing whether parental mindsets and home-learning activities differ across the United States and Denmark. Given the lack of prior cross-cultural investigation of parental mindset, we did not set forth *a priori* hypotheses for potential differences across US and Danish parents. However, we speculated that Danish parents may engage in fewer home-learning activities given that Danish parents report having far less time with their children than US parents. Next, we investigated the associations between parental mindset and home-learning activities. We hypothesized that parents with growth-oriented mindsets toward their children’s abilities and efforts may engage in more home-learning activities, irrespective of cultural context. Last, we investigated country as a moderator of the link between parental mindset and home-learning activities to test if these relations differ by culture.

## Materials and Methods

### Participants

Participants were recruited separately from United States and Denmark upon meeting the eligibility criterion of having at least one child between 3 and 5 years of age. Participant recruitment and participation occurred in parallel. First, the Denmark participants were recruited from a national online panel with ∼200,000 panelists, which is hosted by Epinion, and were contacted via mail. The US participants were recruited via e-mail, postcards, and in-person invitations from a pool of parents of children who participated in recent research projects and parents whose child attend a local preschool in a midwestern state. The final sample consisted of a total of 497 parents (325 Danish and 172 United States) who completed the self-administered online or paper questionnaire, with the same general content but written in each country’s official language. Although we altered wordings of few response items to fit each cultural context (e.g., parental educational attainment), the majority of the questions contained identical information. The descriptive statistics of focal variables by country including comparison statistics are provided in Table 1.

**TABLE 1:** Descriptive statistics and comparison of focal variables by country.

	**Denmark^a^**	**United States^b^**	***t* or χ^2 c^**
		
	***M* (*SD*) or %**	***M* (*SD*) or %**	
**Parental mindset**			
Ability mindset	5.05 (0.71)	5.15 (0.73)	1.54
Effort mindset	5.28 (0.75)	5.34 (0.55)	0.99
**Home-learning activities**			
Family learning activities	2.66 (0.46)	3.03 (0.51)	8.07***
Learning extensions	2.13 (1.41)	3.04 (1.59)	6.55***
Parental time investment	3.26 (0.93)	4.77 (1.04)	16.55***
Parental school involvement	3.10 (1.29)	2.59 (1.49)	−3.92***
**Covariates**			
Parent age	35.87 (5.79)	34.10 (6.58)	−3.09**
Parent education^d^	5.40 (1.45)	4.42 (2.13)	
**Parent occupational status**			
Employed full-time	76.85	61.05	14.05**
Employed part-time	8.95	13.37	
Not employed	14.20	25.58	
**Parent marital status**			
Married	69.14	55.56	66.96***
Cohabiting	24.38	10.53	
Other	6.48	33.92	
Formal childcare	98.15	58.68	132.63***

*SD, standard deviation. ^*a*^n = 324*, *^*b*^n = 172*, *^*c*^t-test statistics are for continuous variables, and χ^2^ are for categorical variables. ^*d*^The scale range was identical but specific response values differed by country.* *p < 0.01 and **p < 0.001.

Whereas the Denmark sample showed a similar gender distribution of the parents (female = 48.77%), the US parent sample was highly female dominant (female = 91.23%). Due to the lack of variance in the US sample, gender was not controlled for the final analyses. Parents’ average age was ∼35 years in both countries. Relatively more parents in the Denmark sample were employed full-time (Denmark = 76.85%; United States = 61.05%), married (Denmark = 69.14%; United States = 55.56%), and cohabiting (Denmark = 24.38%; United States = 10.53%) compared to the US sample. Whereas the majority of the Danish parents were using formal child-care services (i.e., childcare by non-relatives that takes place in either residential or non-residential school-like settings, 98.15%), a higher percentage of US parents were providing parental care (30.54%) as their primary childcare.

### Measures

#### Parental Mindset

We captured two parental mindsets: “ability mindset” and “effort beliefs.” The *ability mindset* scale was based on Dweck’s (1999) Theories of Intelligence (TOI) scale, which distinguishes individuals’ view of intelligence as a trait that is fixed and stable vs. malleable and improvable. For this study, we used selected items from Andersen and Nielsen’s (2016) scale, which was originally based on the study of Muenks et al. (2015) that developed and validated measures of parents’ specific beliefs about the fixedness of their child’s math and verbal abilities. Andersen and Nielsen (2016) further adapted their measure to fit parents of primary school children. Our final measure consisted of the following four items: “After a certain point in childhood, my child’s ability to learn how to read cannot improve; *My child can always improve their ability to learn how to read, no matter how old they are; My child’s ability to learn how to read can only be substantially improved during a specific period in their development;* My child is past the age at which he/she can substantially improve their ability to learn how to read.” We reverse coded some items to represent the extent to the parents think their child’s ability can improve.

The *effort mindset* scale was based on Blackwell et al. (2007), who developed measures to capture youth’s belief that efforts lead to positive outcomes. We used selected items from the measure and adapted them to fit the perspective of the parents’ regarding their preschool child. The final scale consisted of the following three items: “*The harder my child works at something, the better they will be at it; To tell the truth, when my child seems to work too hard at learning activities, it makes me feel like they are not very smart; If my child is not good at learning activities, working hard won’t make them good at it.*” We reverse coded some items to represent the extent to parents think their child’s efforts lead to positive outcomes.

Answers for all the items in both the ability mindset and effort mindset scales ranged from 1 (*strongly disagree*) to 6 (*strongly agree*), and we used the mean values of the items in each scale for our final analyses (ability mindset α = 0.62; and effort mindset α = 0.61).

#### Home-Learning Activities

Home-learning activities consisted of four measures: (a) family learning activities, (b) learning extensions, (c) parental time investment, and (d) parental school involvement. All four measures were adopted from the Early Childhood Longitudinal Study: Kindergarten Class of 2010–2011 (ECLS-K:2011), a longitudinal study that followed a sample of children from kindergarten through the fifth grade to examine topics such as child development and early school experiences. While ECLS-K collected data from several sources including parents and classroom teachers, we only used the parent questionnaire developed for the Kindergarten Year (2010–2011) as they fit the child’s developmental period of our interest. For some scales, we did not include items that were deemed irrelevant to our focal interests (e.g., for the parental school involvement scale, “*have you or other adults in the family gone to a meeting of a parent advisory group or policy council?*”).

*Family learning activities* consisted of 10 items on how often family members engage in activities with their child such as telling stories, singing songs, doing science projects, playing sports, or exercising together, among others, with answers ranging from 1 (*not at all*) to 4 (*every day*). We used the mean value for final analyses (α = 0.82). *Learning extensions* were made up of six items asking whether or not anyone in the family has engaged in the suggested six learning extensions including visiting the library and bookstores (α = 0.53). For our final analyses, we used the sum of six items. *Parental time investment* was based on a question asking how much time family members at home spend playing with their child, ranging from 1 (*no time*) to 6 (*more than 3 h*). Since the same question was asked for both a typical school and weekend day, we used the mean value of the two items given their high correlation (*r* = 0.72, *p* < 0.001; α = 0.83). For *parental school involvement*, we used five items regarding whether or not adults in the household has done each of the five activities including attending an open house and serving as a volunteer in school (α = 0.65). The sum of the five items was used for our final analyses.

#### Covariates

Covariates in this study include parental age, education level, occupational status (i.e., full-time, part-time, and not employed), and marital status (i.e., married, cohabiting, and other). The parent education measure was an ordinal measure, and the specific values differed by country based on their cultural context [e.g., United States = 3 (*some college experience*), 6 (*some postgraduate experience*), 9 (*MD, Law, Ph.D., or other advanced degree*); Denmark = 2 (*less than high school*), 4 (*vocational or technical school*), 6 (*BA, professional BA*), and 8 (*MD, Law, Ph.D., or other advanced degree*)]. We also controlled for a dichotomous indicator of whether the child was involved in formal childcare (i.e., childcare by non-relatives that takes place in either residential or non-residential school-like settings) as opposed to parental care and other informal childcare (i.e., childcare by relatives or less frequently by a non-relative taking place in residential home).

### Analysis Plan

All analyses were conducted in STATA 13. The first research aim comparing the levels of parental mindset and home-learning activities was examined using *t*-tests to indicate whether there are significant differences in the means of each measure by country. We also conducted ordinary least squared (OLS) linear regression regressing parental mindset on the country indicator and all covariates. For the second research aim investigating the relationships between parental mindset and home-learning activities, we conducted OLS linear regression predicting each home-learning activity from parental mindsets and covariates using both the US and Danish samples and controlling for the country indicator (i.e., Denmark = 1). The last research aim investigated the moderating role of country on the associations between parental mindset and home-learning activities, which was tested by including the interaction terms of the county indicator and two parental mindset scales into the direct effect models. For significant interaction effects, we further examined the simple slopes of the association between parental mindset and home learning activities by country.

## Results

### Cross-Cultural Comparison of Parental Mindset and Home-Learning Activities

The results for the first research question comparing the levels of parental mindset and home-learning activities across countries are shown in the right column of Table 1. Our results show that there were no cross-cultural differences in the levels of ability mindset [*t*(494) = 1.54, *p* = 0.125] or effort mindset [*t*(494) = 0.99, *p* = 0.323] between Denmark and the United States. However, as we speculated, US parents were engaging in higher levels of home-learning activities, including family learning activities [*t*(494) = 8.07, *p* < 0.001], learning extension [*t*(494) = 6.55, *p* < 0.001], and parental time investment [*t*(494) = 16.55, *p* < 0.001] compared to the Danish parents. One exception was parental school involvement, which was higher among the Danish parents than the US parents [*t*(494) = 3.92, *p* < 0.001]. We also regressed the parental mindset measures on the country indicator as shown in Table 2, and the results indicate that neither the country indicator nor any of the covariates significantly predicted the parental mindset measures.

**TABLE 2:** Predicting parental mindset from country and covariates.

	**Ability mindset**	**Effort mindset**
	***b* (*SE*)**	***b* (*SE*)**
Denmark sample	−0.14 (0.08)	−0.04 (0.08)
**Covariates**		
Parent age	0.01 (0.01)	0.00 (0.01)
Parent education^a^	0.00 (0.02)	0.03 (0.02)
**Parent work status (ref = not employed)**		
Employed full-time	0.08 (0.09)	−0.09 (0.09)
Employed part-time	0.21 (0.12)	0.08 (0.12)
**Parent marital status (ref = single)**		
Married	−0.04 (0.10)	−0.01 (0.10)
Cohabiting	−0.00 (0.12)	−0.01 (0.11)
Formal childcare	0.04 (0.11)	−0.11 (0.11)
*R*^2^	0.018	0.016

**SE, standard error; Ref, reference group. ^*a*^The scale range was identical, but specific response values differed by country.**

### Association Between Parental Mindset and Home-Learning Activities

Tables 3–5 display the results of the second research aim investigating the universal association between parental mindset and home-learning activities across both cultures. First, Table 3 displays the correlation of focal variables of each country, respectively. According to the results, parental ability mindset was not associated with majority of the home-learning activities in both Denmark and the United States. One exception was the positive and significant correlation between parental ability mindset and time investment among the US parents (*r* = 0.16, *p* = 0.038). Parental effort mindset was not significantly correlated with any of the home-learning activities, but only among the US parents. In contrast, although these correlations are small in nature, parental effort mindset was positively correlated with their family learning activities (*r* = 0.16, *p* = 0.004) and parental time investment (*r* = 0.12, *p* = 0.026) among the Danish parents.

**TABLE 3:** Correlation between main variables.

**Denmark/United States**	**1**	**2**	**3**	**4**	**5**	**6**
(1) Ability mindset		0.34***	0.04	0.11	0.16*	0.01
(2) Effort mindset	0.43***		0.07	–0.00	0.09	0.05
(3) Family learning activities	0.02	0.16**		0.44***	0.51***	0.16*
(4) Learning extensions	–0.10	–0.01	0.35***		0.18*	0.21**
(5) Parental time investment	–0.02	0.12*	0.41***	0.09		–0.05
(6) Parental school involvement	–0.07	–0.04	0.15**	0.19***	0.01	

The diagonal bottom left represents results based on the Denmark sample, and the diagonal upper right represents results based on the US sample. *p < 0.05, **p < 0.01, ***p < 0.001.

The regression results predicting parental mindset from home-learning activities across both cultures with covariates included are shown in Tables 4, 5. According to the results, parental ability mindset was not associated home learning activities, whereas parental effort mindset was positively and significantly associated with their engagement in family learning activities (*b* = 0.09, *SE* = 0.03, *p* = 0.005) and parental time investment (*b* = 0.16, *SE* = 0.07, *p* = 0.016). Furthermore, when examining our covariates, being in the Denmark sample was the most common predictor and was negatively associated with family learning activities (*b* = −0.37, *SE* = 0.06, *p* < 0.001), learning extensions (*b* = −1.19, *SE* = 0.17, *p* < 0.001), and parental time investment (*b* = −1.41, *SE* = 0.11, *p* < 0.001), and positively predicted parental school involvement (*b* = 0.52, *SE* = 0.17, *p* < 0.001).

**TABLE 4:** Predicting family learning activities and learning extensions from parental mindset.

	**Family learning activities**		**Learning extensions**	
	**Model 1**	**Model 2**	**Model 1**	**Model 2**
	***b* (*SE*)**	***b* (*SE*)**	***b* (*SE*)**	***b* (*SE*)**
**Parental mindset**				
Ability mindset		−0.01 (0.03)		−0.09 (0.10)
Effort mindset		0.09 (0.03)**		0.01 (0.10)
**Covariates**				
Parent age	−0.00 (0.00)	−0.00 (0.00)	0.01 (0.01)	0.01 (0.01)
Parent education^a^	0.01 (0.01)	0.01 (0.01)	0.03 (0.04)	0.03 (0.04)
**Parent work status (ref = not employed)**				
Employed full-time	−0.12 (0.06)*	−0.11 (0.06)	0.08 (0.19)	0.09 (0.19)
Employed part-time	−0.18 (0.08)*	−0.18 (0.08)*	−0.39 (0.26)	−0.37 (0.26)
**Parent marital status (ref = other)**				
Married	0.12 (0.07)	0.12 (0.07)	0.26 (0.21)	0.26 (0.21)
Cohabiting	0.02 (0.08)	0.02 (0.08)	0.01 (0.24)	0.01 (0.24)
Formal childcare	−0.00 (0.07)	0.01 (0.07)	0.34 (0.22)	0.34 (0.22)
Denmark sample	−0.37 (0.06)***	−0.37 (0.06)***	−1.18 (0.17)***	−1.19 (0.17)***
*R*^2^	0.141***	0.156***	0.112***	0.114***

SE, standard error; Ref, reference group. ^*a*^The scale range was identical but specific response values differed by country. *p < 0.05, **p < 0.01, ***p < 0.001.

**TABLE 5:** Predicting parental time investment and parental school involvement from parental mindset.

	**Parental time investment**		**Parental school involvement**	
	**Model 1**	**Model 2**	**Model 1**	**Model 2**
	***b* (*SE*)**	***b* (*SE*)**	***b* (*SE*)**	***b* (*SE*)**
**Parental mindset**				
Ability mindset		0.00 (0.07)		−0.11 (0.10)
Effort mindset		0.16 (0.07)*		−0.03 (0.10)
Covariates				
Parent age	−0.03 (0.01)***	−0.03 (0.01)***	0.01 (0.01)	0.01 (0.01)
Parent education^a^	−0.02 (0.03)	−0.03 (0.03)	0.05 (0.04)	0.05 (0.04)
**Parent work status (ref = not employed)**				
Employed full-time	−0.06 (0.12)	−0.04 (0.12)	−0.21 (0.17)	−0.20 (0.17)
Employed part-time	−0.16 (0.17)	−0.17 (0.17)	−0.20 (0.24)	−0.17 (0.24)
**Parent marital status (ref = other)**				
Married	0.14 (0.13)	0.14 (0.13)	0.26 (0.19)	0.25 (0.19)
Cohabiting	0.14 (0.16)	0.14 (0.16)	−0.08 (0.23)	−0.08 (0.23)
Formal childcare	−0.20 (0.14)	−0.18 (0.14)	−0.19 (0.22)	−0.19 (0.22)
Denmark sample	−1.42 (0.11)***	−1.41 (0.11)***	0.54 (0.17)**	0.52 (0.17)**
*R*^2^	0.390***	0.399***	0.053**	0.057**

SE, standard error; Ref, reference group. ^*a*^The scale range was identical but specific response values differed by country. *p < 0.05, **p < 0.01, ***p < 0.001.

### Cultural Context as a Moderator of the Association Between Parental Mindset and Home-Learning Activities

**FIGURE 1:**
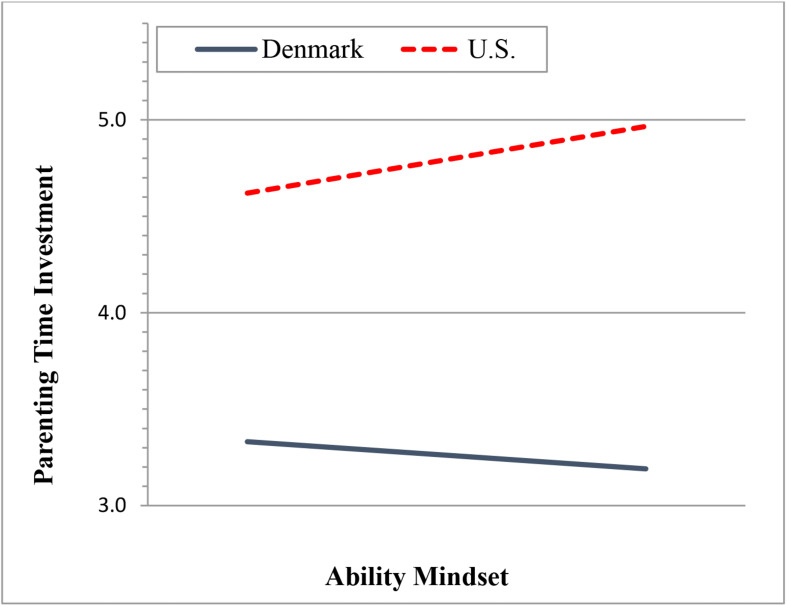
Association between parental ability mindset and parental time investment by country.

To address the third research question investigating the moderating role of cultural context on the link between parental mindset and home-learning activities, we regressed home-learning outcomes on the interaction terms of each parental mindset variable and the Denmark indicator. First, we found that county did not moderate the relationship between parental effort mindset and home-learning activities (results not shown in table). However, the country indicator was found to moderate the association between parental ability mindset and parental time investment (see Table 6). Specifically, the association between parental ability mindset and time investments was significantly weaker for Danish parents compared to the US parents (*b* = −0.29, *SE* = 0.13, *p* = 0.029). The simple slopes in Figure 1 show that for Danish parents, ability mindset was not associated with their parental time investment (*b* = −0.10, *SE* = 0.08, *p* = 0.212), whereas US parents showed a marginal and positive association between ability mindset and time investment (*b* = 0.24, *SE* = 0.12, *p* = 0.052).

**TABLE 6:** Predicting home-learning activities from interaction of country and ability mindset.

	**Family learning activities**	**Learning extensions**	**Parental time investment**	**Parental school involvement**
	***b* (*SE*)**	***b* (*SE*)**	***b* (*SE*)**	***b* (*SE*)**
**Parental mindset**				
Ability mindset	0.01 (0.05)	0.18 (0.17)	0.19 (0.11)	−0.09 (0.16)
Effort mindset	0.10 (0.03)**	0.04 (0.11)	0.18 (0.07)**	−0.03 (0.10)
**2-way interaction terms**				
Ability mindset*Denmark	−0.04 (0.07)	−0.40 (0.20)	−0.29 (0.13)*	−0.03 (0.19)
Covariates				
Parent age	0.00 (0.00)	0.01 (0.01)	−0.03 (0.01)***	0.01 (0.01)
Parent education^a^	0.01 (0.01)	0.03 (0.04)	−0.03 (0.03)	0.05 (0.04)
**Parent work status (ref = not employed)**				
Employed full-time	−0.11 (0.06)	0.09 (0.19)	−0.04 (0.12)	−0.20 (0.18)
Employed part-time	−0.19 (0.08)*	−0.39 (0.26)	−0.19 (0.17)	−0.17 (0.24)
**Parent marital status (ref = other)**				
Married	0.12 (0.07)	0.26 (0.21)	0.15 (0.13)	0.25 (0.19)
Cohabiting	0.02 (0.08)	0.01 (0.24)	0.14 (0.15)	−0.08 (0.23)
Formal childcare	0.01 (0.07)	0.34 (0.22)	−0.18 (0.14)	−0.19 (0.22)
Denmark sample	−0.19 (0.34)	0.85 (1.06)	0.05 (0.68)	0.68 (0.99)
*R*^2^	0.156***	0.121***	0.405***	0.057**

SE, standard error; Ref, reference group. ^*a*^The scale range was identical but specific response values differed by country. *p < 0.05, **p < 0.01, ***p < 0.001.

## Discussion

The present study adds to an emerging research base concerning the role of parental mindset in shaping behaviors and is a unique contribution to this base given our focus on the relations between parental mindset and home-learning activities for US and Danish parents. Given the importance of parental beliefs to their children’s development (Curenton and Justice, 2008; Andersen and Nielsen, 2016; Davis et al., 2016), it is necessary to learn more about mindset as a particular dimension of parental beliefs, as it may influence their behaviors toward their children. We speculated that caregivers whose mindset is more growth-oriented may provide their children with more frequent home-learning opportunities, as caregivers with a growth-oriented mindset would view these opportunities as catalysts for child development, as suggested by recent intervention research (Rowe and Leech, 2018). We further speculated that caregivers whose belief system values the role of effort in development and learning would provide their children with more frequent home-learning opportunities as well. In addition, we also explored whether there is variability in caregiver mindset cross-culturally by comparing parental mindset in a culture with relatively modest investments in social welfare, including childcare provision, and one with considerable welfare investments including universal childcare.

We highlight several major contributions of this study. First, we found that for our full sample of parents, parents’ effort mindset, but not ability mindset, was positively associated with the frequency of family learning activities and parental time investment. Specifically, parents whose belief system values the role of effort in learning tended to engage in more learning activities with their children, such as telling stories and sharing books; in addition, they reported to spend more time with their children overall. This finding represents a unique contribution to the literature on mindset, much of which has focused on the relations between students’ mindsets and their academic achievement (Sisk et al., 2018). Although the average correlation between mindset and achievement is very modest, based on recent meta-analytic methods (Sisk et al., 2018), mindset can be easily modified and thus offers a potential valuable mechanism to improve students’ efforts and, in turn, their achievement over time. The present study is one of the first to explore the relations between parents’ mindsets toward their children and engagement in home-learning activities and provide a potentially viable approach toward enhancing home-learning opportunities for children via mindset-focused interventions.

Results of the present work suggest, potentially, that increasing parents’ effort beliefs may result in increased learning activities in the home environment, although this needs to be tested causally. Some support for this conjecture is derived from a recent experimental study by Rowe and Leech (2018), who embedded training on parental mindset into a language-focused intervention delivered to parents of toddlers. These researchers found that parents’ language-facilitation behaviors improved most prominently for parents with fixed mindsets at study start. Perhaps, this is because parents with a growth-oriented mindset already engage in significant home-learning activities with their children, buffering the effects of the intervention, whereas those with a fixed mindset can be induced to improve home-learning opportunities by modifying their beliefs about their children’s abilities and efforts.

One might question why parents’ effort mindset, rather than ability mindset, was associated with parents’ home-learning opportunities, including time investments with their children. Recall that effort mindset captures parents’ beliefs in the role of effort as influential to learning, whereas ability mindset captures parents’ beliefs about the malleability of their children’s skills. We speculate that parents’ effort mindset toward their children may reflect their beliefs about effort more generally; specifically, parents who believe that effort is important may therefore be included to direct effort toward their children’s learning in the home environment. If this is the case, it would explain why parents who value effort would direct more effort toward their children in terms of provision of family-learning activities and actual time investments.

The second contribution that we seek to emphasize concerns our focus on potential cross-cultural differences in parental mindset across our US and Danish samples. As we discuss in the introduction, these two cultural contexts vary in key ways that might lead to differences in parental mindset and provision of home-learning activities; these include distinctions between the countries in terms of societal inequality, adults’ labor-force participation, parents’ time investments with their children, and access to childcare. Our cross-cultural study had several interesting findings. First, the samples were quite distinct in several compelling but unexpected ways. Although all parents recruited into the study had children of preschool age, the US parents were slightly younger, less likely to be cohabiting, more likely to be employed part-time, and less likely to be using formal childcare compared to the Danish parents. In addition, the US parents reported higher rates of family learning activities, learning extensions, and time investment compared to Danish parents. This was to be expected based on evidence showing that Danish parents’ time investments with their children tends to be lower than parents in other countries, including the United States (Craig and Mullan, 2012), possibly because of their high level of access to childcare and high rates of labor-force participation by parents. Danish parents did report more school involvement than US parents, perhaps in part because preschool attendance is more common in Denmark and because parents are more likely to enroll their children in preschool at earlier ages in Denmark, which in turn, may facilitate parent involvement and parent–teacher relationships. For example, according to the most recent OECD data for each country, 56% of Danish children ages 0–2 are enrolled in childcare (in 2017), compared to only 28% of US children (in 2011: Organisation for Economic Co-operation and Development, 2020b). Interestingly, however, we found no meaningful difference between US and Danish parents with respect to their mindset: both sets of parents held similar perspectives regarding ability mindset and effort mindset, indicating that these types of parental beliefs are not conditioned on cross-cultural differences. It is interesting to find that despite distinct differences across these cultures in parent time investments with their children and labor-force participation (Craig and Mullan, 2012), parents hold similar beliefs regarding ability and effort.

A finding of additional note concerns the interplay among country (United States and Denmark), parents’ effort mindset, and parental time investment. Although it is established that Danish parents tend to spend less time with their children than parents in other countries (Craig and Mullan, 2012), the present findings show an interesting phenomenon: namely, a positive relationship between US parents’ effort mindset and time investments with their children, yet the absence of such a relationship for the Danish parents. This phenomenon may reflect the limited time Danish parents have with their children, as shown specific to our sample but also in larger samples of Danish parents compared to parents in other settings. For instance, Craig and Mullan (2012) showed that for partnered mothers, Danish mothers reported spending ∼3 h fewer caring for their young children compared to US and Australian mothers. This being the case, the limited time availability of Danish moms to care for their children may serve to dampen the relations between effort mindset and time investments. This suggests that in some circumstances, efforts to modify parents’ mindset toward their children may offer limited benefits in terms of the home-learning environment; further research should examine whether parental mindset shapes decisions about children’s out-of-home care in contexts where dual parental employment is high. However, in other circumstances, such as the United States, helping parents to develop a stronger orientation toward the role of effort in learning may lead to heightened time investments with their children as a mechanism for improved child outcomes.

We highlight several limitations of this work as well as future research directions. First, we relied on parent report for measures of parental mindset and home-learning opportunities, and unfortunately, our measures of parental mindset showed relatively low reliabilities. This work would have been strengthened with direct observations of home-learning opportunities and alternative approaches to assessing parental mindset. Likewise, there is a need for more methodological work to be conducted in developing mindset questionnaires with increased reliability. Relatedly, with the reliance on parent report for all measurements in this study, we did not address measurement invariance among the sample. Second, our samples of US and Danish parents differed in key ways, possibly due to differences in ascertainment activities. Future work should use identical ascertainment activities if possible. Despite these limitations, the present work provides evidence that parental mindset corresponds to the volume of home-learning activities that they provide to their young children, paving the way for additional work on this topic. We propose several important lines of work. First, there is a need for causally interpretable studies that assess whether mindset-focused interventions delivered to parents of young children improves the home-learning environment. Second, there is a need for further cross-cultural work to determine whether parental mindset is a universal phenomenon of importance and how cultural contexts may contribute to mindset. Collectively, such efforts can lead to fundamental improvements in the mindset construct and its relevance to parenting and child development in global contexts.

## Data Availability Statement

The raw data supporting the conclusions of this article will be made available by the authors, without undue reservation, to any qualified researcher.

## Ethics Statement

The studies involving human participants were reviewed and approved by The Ohio State University and Aarhus University. The patients/participants provided their written informed consent to participate in this study.

## Author Contributions

LJ, DB, and KP conceptualized the overarching goals of the study. DB and KP wrote funding proposals and managed data collection and data entry at two sites. SC, DB, and KP researched and developed the questionnaires for the study. SC was engaged in direct data collection at one site, conducted data analyses, and wrote much of the methods and results. LJ wrote much of the literature review and discussion. LJ, DB, KP, and SC reviewed and edited the entire draft. All authors contributed to the article and approved the submitted version.

## Conflict of Interest

The authors declare that the research was conducted in the absence of any commercial or financial relationships that could be construed as a potential conflict of interest.

## References

[B1] Andersen S. C. Nielsen H. S. (2016). Reading intervention with a growth mindset approach improves children’s skills. *Proc. Natl. Acad. Sci. U.S.A.* 113 12111–12113. 10.1073/pnas.1607946113 27729533 PMC5087073

[B2] Armstrong M. I. Birnie-Lefcovitch S. Ungar M. T. (2005). Pathways between social support, family well being, quality of parenting, and child resilience: what we know. *J. Child Family Stud.* 14 269–281. 10.1007/s10826-005-5054-4

[B3] Baker M. Milligan K. (2016). Boy-girl differences in parental time investments: evidence from three countries. *J. Hum. Capital* 10 399–441. 10.1086/688899

[B4] Blackwell L. S. Trzesniewski K. H. Dweck C. S. (2007). Implicit theories of intelligence predict achievement across an adolescent transition: a longitudinal study and an intervention. *Child Dev.* 78 246–263. 10.1111/j.1467-8624.2007.00995.x 17328703

[B5] Bono E. D. Francesconi M. Kelly Y. Sacker A. (2016). Early maternal time investment and early child outcomes. *Econ. J.* 126 F96–F135.

[B6] Breit-Smith A. Cabell S. Q. Justice L. M. (2010). Home literacy experiences and early childhood disability: a descriptive study using the national household education surveys (NHES) program database. *Lang. Speech Hear. Serv. Sch.* 41 96–107. 10.1044/0161-1461(2009/08-0048) 20051581

[B7] Bronfenbrenner U. (1994). Ecological models of human development. *Read. Dev. Child.* 2 37–43.

[B8] Central Intelligence Agency, (2020). *Country Comparison : Distribution Of Family Income - Gini Index. The World Factbook.* Available at: https://www.cia.gov/library/publications/the-world-factbook/rankorder/2172rank.html (accessed March 2, 2020).

[B9] Christian K. Morrison F. J. Bryant F. B. (1998). Predicting kindergarten academic skills: interactions among child care, maternal education, and family literacy environments. *Early Child. Res. Q.* 13 501–521. 10.1016/s0885-2006(99)80054-4

[B10] Craig L. Mullan K. (2012). Lone and partnered mothers’ childcare time within context in four countries. *Eur. Soc. Rev.* 28, 512–526.

[B11] Curenton S. M. Justice L. M. (2008). Children’s preliteracy skills: influence of mothers’ education and beliefs about shared-reading interactions. *Early Educ. Dev.* 19 261–283. 10.1080/10409280801963939

[B12] Davis H. S. Gonzalez J. E. Pollard-Durodola S. Saenz L. M. Soares D. A. Resendez N. (2016). Home literacy beliefs and practices among low-income Latino families. *Early Child Dev. Care* 186 1152–1172. 10.1080/03004430.2015.1081184

[B13] Dearing E. Kreider H. Weiss H. B. (2008). Increased family involvement in school predicts improved child–teacher relationships and feelings about school for low-income children. *Marriage Fam. Rev.* 43 226–254. 10.1080/01494920802072462

[B14] Duncan G. J. Ziol-Guest K. M. Kalil A. (2010). Early-childhood poverty and adult attainment, behavior, and health. *Child Dev.* 81 306–325. 10.1111/j.1467-8624.2009.01396.x 20331669

[B15] Dweck C. S. (1999). Caution–praise can be dangerous. *Am. Educ.* 23, 4–9.

[B16] Evans G. W. Kim P. (2013). Childhood poverty, chronic stress, self-regulation, and coping. *Child Dev. Perspect.* 7 43–48. 10.1111/cdep.12013

[B17] Fishel M. Ramirez L. (2005). Evidence-based parent involvement interventions with school-aged children. *Sch. Psychol. Q.* 20 371–402. 10.1521/scpq.2005.20.4.371

[B18] Gonzalez J. E. Taylor A. B. McCormick A. S. Villareal V. Kim M. Perez E. (2011). Exploring the underlying factor structure of the home literacy environment (HLE) in the English and Spanish versions of the familia inventory: a cautionary tale. *Early Child. Res. Q.* 26 475–483. 10.1016/j.ecresq.2010.12.001

[B19] Heiland F. Price J. Wilson R. (2017). Maternal employment and time investments in children. *Rev. Econ. Househ.* 15 53–67. 10.1007/s11150-014-9278-1

[B20] Justice L. Skibbe L. E. McGinty A. S. Piasta S. B. Petrill S. (2011). Feasibility, efficacy, and social validity of home-based storybook reading intervention for children with language impairment. *J. Speech Lang. Hear. Res.* 54 523–538. 10.1044/1092-4388(2010/09-0151) 20719873 PMC12416296

[B21] Kalil A. Ryan R. Chor E. (2014). Time investments in children across family structures. *Ann. Am. Acad. Polit. Soc. Sci.* 654 150–168. 10.1177/0002716214528276

[B22] Korat O. Klein P. Segal-Drori O. (2007). Maternal mediation in book reading, home literacy environment, and children’s emergent literacy: a comparison between two social groups. *Read. Writ.* 20 361–398. 10.1007/s11145-006-9034-x

[B23] Lederer S. H. (2001). Efficacy of parent-child language group intervention for late-talking toddlers. *Infant Toddler Intervention* 11 223–236.

[B24] Mammen K. (2011). Fathers’ time investments in children: do sons get more? *J. Popul. Econ.* 24 839–871. 10.1007/s00148-009-0272-5

[B25] Manolitsis G. Georgiou G. K. Tziraki N. (2013). Examining the effects of home literacy and numeracy environment on early reading and math acquisition. *Early Child. Res. Q.* 28 692–703. 10.1016/j.ecresq.2013.05.004

[B26] Muenks K. Miele D. B. Ramani G. B. Stapleton L. M. Rowe M. L. (2015). Parental beliefs about the fixedness of ability. *J. Appl. Dev. Psychol.* 41, 78–89.

[B27] Neidell M. J. (2000). *Early Parental time Investments in Children’s Human Capital Development: Effects of time in the First year on Cognitive and Non-Cognitive Outcomes.* UCLA Department of Economics Working Paper, 806 Los Angeles, CA: University of California.

[B28] Niklas F. Cohrssen C. Tayler C. (2016). Improving preschoolers’ numerical abilities by enhancing the home numeracy environment. *Early Educ. Dev.* 27 372–383. 10.1080/10409289.2015.1076676

[B29] Organisation for Economic Co-operation and Development (2015). *In It Together: Why Less Inequality Benefits All.* Paris: OECD.

[B30] Organisation for Economic Co-operation and Development (2020a). *Family Database.* Available online at: https://stats.oecd.org/Index.aspx?DataSetCode=FAMILY (accessed March 2, 2020).

[B31] Organisation for Economic Co-operation and Development (2020b). *Income Inequality.* Available online at: https://data.oecd.org/inequality/income-inequality.htm (accessed March 2, 2020).

[B32] Roberts M. Y. Kaiser A. P. (2011). The effectiveness of parent-implemented language interventions: a meta-analysis. *Am. J. Speech Lang. Pathol.* 20 180–199. 10.1044/1058-0360(2011/10-0055) 21478280

[B33] Rowe M. L. (2008). Child-directed speech: relation to socioeconomic status, knowledge of child development and child vocabulary skill. *J. Child Lang.* 35 185–205. 10.1017/s0305000907008343 18300434

[B34] Rowe M. L. Leech K. A. (2018). A parent intervention with a growth mindset approach improves children’s early gesture and vocabulary development. *Dev. Sci.* 22:e12792. 10.1111/desc.12792 30570813 PMC7041843

[B35] Sayer L. C. Gornick J. C. (2012). Cross-national variation in the influence of employment hours on child care time. *Eur. Sociol. Rev.* 28 421–442. 10.1093/esr/jcr008

[B36] Sisk V. F. Burgoyne A. P. Sun J. Butler J. L. Macnamara B. N. (2018). To what extent and under which circumstances are growth mind-sets important to academic achievement? Two meta-analyses. *Psychol. Sci.* 29 549–571. 10.1177/0956797617739704 29505339

[B37] Wagner M. Spiker D. Linn M. I. (2002). The effectiveness of the Parents as Teachers program with low-income parents and children. *Topics Early Child. Spec. Educ.* 22 67–81. 10.1177/02711214020220020101

[B38] World Bank (2020). *GINI Index (World Bank estimate) – United States.* Available at: https://data.worldbank.org/indicator/SI.POV.GINI?locations=US (accessed March 2, 2020).

